# A two-sample Mendelian randomization study of circulating lipids and deep venous thrombosis

**DOI:** 10.1038/s41598-023-34726-3

**Published:** 2023-05-08

**Authors:** Pan Luo, Qiling Yuan, Xianjie Wan, Mingyi Yang, Peng Xu

**Affiliations:** Department of Joint Surgery, HongHui Hospital, Xi’an Jiaotong University, Xi’an, 710054 Shaanxi China

**Keywords:** Genetics, Diseases

## Abstract

In view of the current debate about the relationship between lipids and deep venous thrombosis (DVT) in clinical studies, a two-sample Mendelian randomization (MR) study was conducted to clarify the effects of five circulating lipids (apolipoprotein A1, apolipoprotein B, low-density lipoprotein, high-density lipoprotein and triglycerides) on DVT from the perspective of genetic inheritance. Five lipids (exposure) were analysed by MR with DVT (outcome) from two different data sources. For the analysis, we used inverse variance weighting and a weighted mode, weighted median, simple mode and MR–Egger regression to analyse the effect of circulating lipids on DVT. In addition, we used the MR–Egger intercept test, Cochran’s Q test and “leave-one-out” sensitivity analysis to evaluate horizontal multiplicity, heterogeneity and stability, respectively, in the analysis. In the analysis, the two-sample Mendelian randomization analysis of five common circulating lipids and DVT showed that common circulating lipids had no causal effect on DVT, which is somewhat inconsistent with the findings of many published observational studies. Based on our results, our two-sample MR analysis failed to detect a statistically significant causal relationship between five common circulating lipids and DVT.

## Introduction

Deep venous thrombosis (DVT) is a common venous thromboembolism that usually affects the lower extremities. The annual incidence rate is close to 1.6 per 1000 people^1^. Thrombosis usually begins in the deep vein of the leg and spreads proximally. The main risk factors for DVT include trauma, cancer and gene mutations that promote blood hypercoagulability^2^. The formation mechanism of DVT can be explained by Virchow’s triad, i.e., a blood flow disorder, a hypercoagulable state of the blood and a procoagulant state of the vascular wall^3^.

At present, some studies suggest that lipid metabolism disorders can affect DVT; although there are many clinical studies on the effect of lipids on venous thrombosis (VT), current conclusions are still controversial. Some studies have suggested that the levels of low-density lipoprotein (LDL), high-density lipoprotein (HDL) and triglycerides (TGs) are not associated with an increased risk of VT, whereas a decrease in apolipoprotein B (APOB) and apolipoprotein A1 (APOA1) levels is associated with an increased risk of VT, but the specific mechanism has not been clarified^4–6^. However, Schouwenburg et al.^7^ found no association between apolipoproteins and the risk of venous thromboembolism. Because lipid levels can be regulated by lifestyle and medications, the relationship between lipid levels and VT and its pathophysiology is a clinical issue worth exploring^8^. A previous study showed that lipid-lowering drugs are associated with a reduced risk of VT, which may indicate the possible role of lipids in the pathophysiology of VT^9^. The ambiguous relationship between blood lipids and DVT requires further clarification.

Mendelian randomization (MR) is a new strategy based on Mendelian inheritance and uses genetic variation as an instrumental variable (IV) to study the causal relationship between different traits^10^. MR provides a valuable tool, especially when randomized controlled trials to check causality are not feasible and observational studies have biased associations due to confounding or reverse causality^11^. MR also enables the use of published results from large genome-wide association studies (GWASs) to study risk factors (exposure) and disease (outcome) and to avoid confounding factors and reverse causality deviations in observational studies^12^. Fernando et al. used MR to analyse the relationship between inflammatory pathways and suicide and found that IL-6 signalling was associated with suicide^13^. In view of the current debate on the relationship between lipids and DVT in clinical studies and the limitations of clinical studies, a two-sample MR study was conducted to clarify the effects of five kinds of circulating lipids (APOA1, APOB, LDL, HDL and TGs) on DVT from a genetic perspective.

## Methods

We referred to Xu et al.^14^ two-sample Mendelian randomization study to test the causal relationship between DVT and circulating lipids.

### Data sources

The DVT data used in this article all came from Neale lab analysis of UK Biobank phenotypes (excluding pulmonary embolism). The DVT data in the analysis included 6767 DVT patients and 330,392 control cases. Up to 10,894,596 SNPs were included in the analysis. Information on various phenotypes was collected from each participant, and blood samples were collected when the subjects visited the UK Biobank Assessment Centre. DNA extraction and genotyping were carried out in the Affymetrix Research Service Laboratory. Summary GWAS data on DVT can be downloaded from the UK Biobank (UKBB) database and MRC IEU OpenGWAS repositories (https://gwas.mrcieua.ac.uk/). UKBB details, including geographic areas, recruitment processes, and other features, have been described in previous articles^15^.

Data on circulating lipids came from two other non-UK biobank studies. APOA1 and APOB data came from the research of Kettunen et al. They conducted an extended genome-wide association study of as many as 24,925 individuals from 10 European studies. Up to 12,133,295 SNPs were included in the meta-analysis after applying quality control filters^16^. GWAS data on LDL, HDL and TGs came from the research of Willer et al. In the study, to identify new loci and extract known loci that affect these blood lipids, they examined 188,578 people using genome-wide and custom genotyping arrays^17^. More details on the sample treatment, determination details, genotyping quality control, staging, interpolation and association tests of circulating lipids (APOA1, APOB, LDL, HDL and TGs) included in this analysis can be obtained from previous reports^16,17^. Details of all the data and GWAS IDs used are shown in Supplementary File 1.

### Selection of instrumental variables

When selecting instrumental variables (IVs), we followed the three basic hypotheses of MR: first, genetic variation should be closely related to the exposure; second, variation should not be affected by confounding factors of the relationship between the exposure and outcome; and third, the exposure should only affect the outcome (i.e., pleiotropy should be eliminated, and the exclusion limitation hypothesis should be satisfied). Therefore, we extracted genomic single-nucleotide polymorphisms (SNPs) associated with exposure (P < 5 × 10^−8^)^14^. In addition, none of the instrumental SNPs were in linkage disequilibrium (LD). We performed the clumping process (R^2^ < 0.001, Magna window size = 10,000 kb) to eliminate the LD between the SNPs^14^. Third, SNPs with a minor allele frequency (MAF) < 0.01 were removed. By default, if the SNP for a particular request did not exist in the resulting GWAS, the SNP (agent) with the requested SNP (target) in the LD was searched^14^. The LD agent was defined using 1000 genomes of European sample data. Then, we searched the human gene phenotypic association database (PhenoScanner V2) to evaluate possible pleiotropic associations between instrument variables and other phenotypes and excluded DVT-related IVs (such as weight and whole-body fat mass). In addition, to test whether there was a weak instrumental deviation in the IV, we used the F statistic (F = R^2^ (n − k − 1)/k (1 − R^2^), where R^2^ is the variance of exposure explained by selected instrumental variables (obtained from the MR Steiger directionality test), n is the sample size, and k is the total variables. If the F statistic of the IV is much greater than 10, it indicates that the possibility of weak instrument variable bias is very small^14^.

### Study design and statistical analyses

We first conducted univariable MR analyses for each lipid-related trait. Because there are varying degrees of overlap between SNPs related to different lipid properties, it may not be accurate to test the effect of individual lipids on DVT. Multivariable MR allows associations of SNPs with multiple phenotypes to be included in the analysis so that the direct impact of each phenotype on the outcome can be estimated^18,19^. Therefore, we regarded multivariable MR as the primary analysis method. A multiple test correction with a *p* value < 0.01 (0.05/5 exposures) was regarded as a significant correlation.

When horizontal pleiotropy does not exist in the analysis, the inverse variance weighted mode can provide the most accurate causal estimation^20^. When there is heterogeneity in the analysis, random effects IVW can provide accurate causal estimation. We also used several other MR methods, including the MR–Egger method, weighted median method, simple mode method and weighted mode method, to test the robustness of the results^21^. We used the MR–Egger method^22^ to analyse the sensitivity and check the consistency and possible pleiotropy of the correlation. The weighted median method provides consistent causal estimates when the effective tool has more than 50% of the weight^23^. MR–Egger regression can detect multiplicity by intercept; however, it impairs statistical power^22^. The effect of horizontal heterogeneity was evaluated and tested by Cochrane’s Q value in the MR–Egger regression and IVW test, with a P value of 0.05 indicating considerable heterogeneity. The odds ratios (ORs) and corresponding 95% confidence intervals (CIs) of lipids were associated with the presence or absence of genetically predicted DVT. We then conducted a further sensitivity analysis using the “leave-one-out” test to evaluate whether the results were affected by individual SNPs. All statistical data analyses were conducted with R (version 4.2.0) software (TwoSampleMR and MendelianRandomization packages).

### Ethical statement

All data were downloaded from the internet.

## Results

### Selection of instrumental variables

The details of all independent SNPs associated with exposure are shown in Supplementary File 2. In our study, the F statistics of the instrumental variables associated with exposure were all greater than 10, indicating that the possibility of variable deviation of weak instrumental variables was very small.

### The causal relationship between APOA1 and DVT

**Figure 1:**
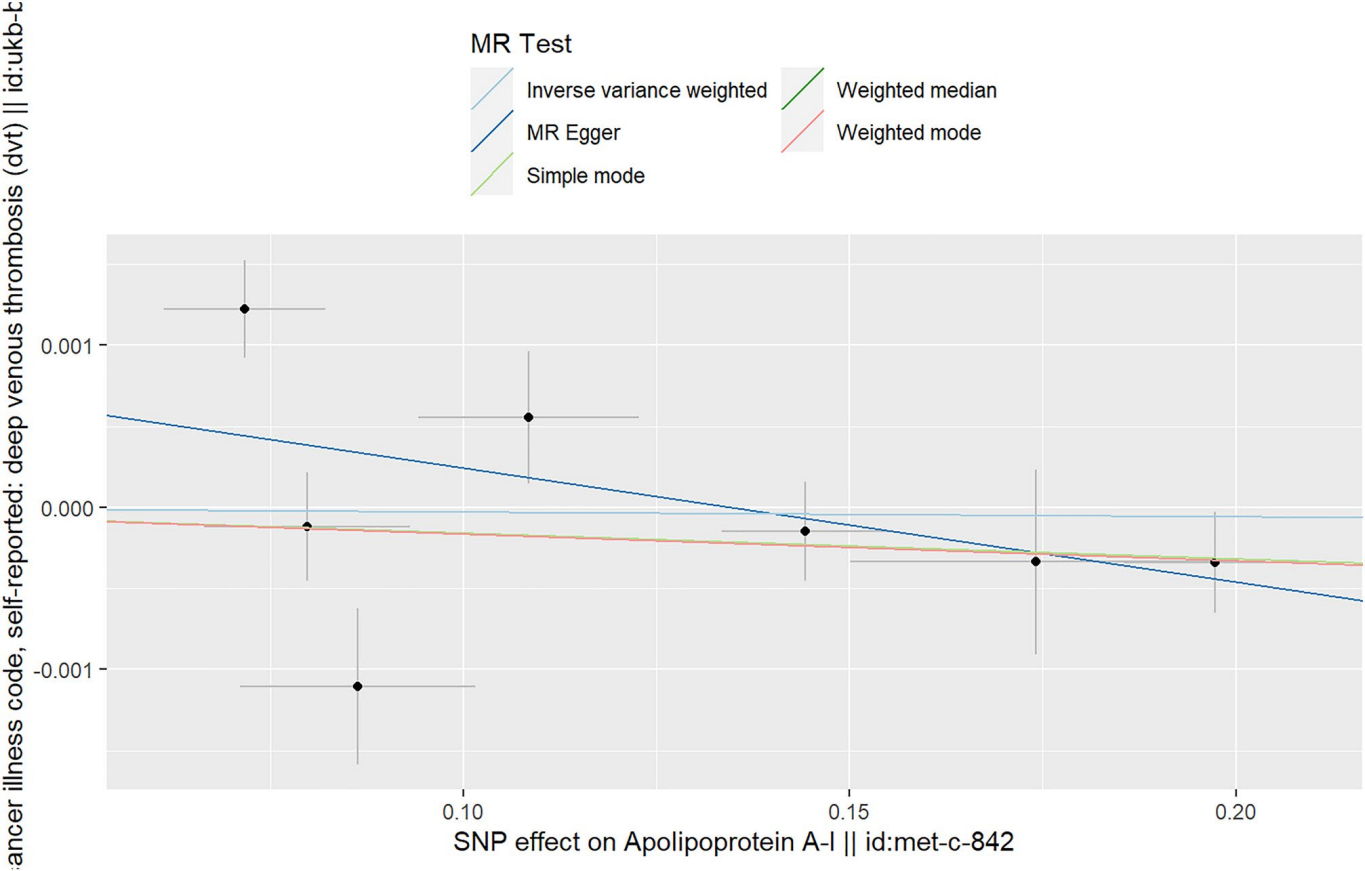
Scatter plot of the causal relationship between APOA1 and DVT. Analyses were conducted using the conventional IVW, weighted median, weighted mode, simple mode and MR‒Egger methods. The slope of each line corresponds to the estimated MR effect per method.

In the MR analyses, we found that there was no causal relationship between APOA1 (exposure) and DVT (outcome) using the various MR analysis methods (IVW (random effects): Beta = − 3e−4, P_beta_ = 0.870; weighted median: Beta = − 0.001, P_beta_ = 0.480; weighted mode: Beta = − 0.001, P_beta_ = 0.447; simple mode: Beta = − 4e−4, P_beta_ = 0.847; MR‒Egger: Beta = − 0.006, P_beta_ = 0.291) (Table 1, Fig. 1). In addition, multivariable MR analysis showed that there was no causal relationship between APOA1 and DVT (Beta = − 0.002, P = 0.701) (Table 3). Because the heterogeneity test in the analysis found that there was a certain level of heterogeneity (IVW: Q–*p* values = 0.002; MR–Egger: Q *p* values = 0.004) (Table 2), we performed an IVW (random effects) analysis, and the results were consistent with those of other models: both showed that there was no causal relationship (Table 1).

**Table 1:** MR estimates from different methods of assessing the causal effect of circulating lipids on DVT.

Exposure-outcome	No. of SNPs	IVW (random effects)			Weighted median		Weighted mode		Simple mode		MR‒Egger	
		Beta (95% CI)	OR (95% CI)	*p* value	Beta (95% CI)	*p* value	Beta (95% CI)	*p* value	Beta (95% CI)	*p* value	Beta (95% CI)	*p* value
APOA1-DVT	10	− 3e−4 (− 0.004, 0.003)	0.999 (0.996, 1.003)	0.870	− 0.001 (− 0.004, 0.002)	0.480	− 0.001 (− 0.004, 0.002)	0.447	− 4e−4 (− 0.004, 0.004)	0.847	− 0.006 (− 0.016, 0.004)	0.291
APOB-DVT	19	− 0.001 (− 0.008, 0.005)	0.998 (0.991, 1.005)	0.715	− 0.004 (− 0.006, − 0.001)	0.005	− 0.006 (− 0.008, − 0.003)	0.003	0.001 (− 0.004, 0.006)	0.669	− 0.008 (− 0.020, 0.003)	0.165
LDL-DVT	69	− 0.002 (− 0.004, 0.001)	0.998 (0.995, 1.001)	0.117	− 0.001 (− 0.004, 0.001)	0.366	− 0.001 (− 0.004, 0.002)	0.637	− 0.001(− 0.007, 0.005)	0.767	− 3e−4 (− 0.004, 0.003)	0.881
HDL-DVT	86	− 5e−4 (− 0.003, 0.002)	0.999 (0.996, 1.002)	0.670	− 0.001 (− 0.004, 0.001)	0.259	− 0.001 (− 0.003, 0.001)	0.484	0.003 (− 0.003, 0.008)	0.384	− 3.54–4 (− 0.004, 0.004)	0.870
TGs-DVT	54	− 0.004 (− 007, − 1.82e−4)	0.997 (0.993, 1.001)	0.038	− 0.004 (− 0.008, − 0.001)	0.017	− 0.003 (− 0.006, − 2.46e−4)	0.038	− 0.003 (− 0.008, 0.002)	0.270	− 0.003 (− 0.008, 0.003)	0.335

*DVT* deep venous thrombosis, *APOA1* apolipoprotein A1, *APOB* apolipoprotein B, *LDL* low-density lipoprotein, *HDL* high-density lipoprotein, *TGs* triglycerides, *Se* standard error, *SNPs* single-nucleotide polymorphisms, *MR* Mendelian randomization, *IVW* inverse variance weighting.

**Table 2:** Heterogeneity tests and MR‒Egger intercept values.

Exposure-outcome	MR‒Egger				IVW	
	Intercept (Se)	Intercept P value	Cochran Q statistics (df)	Q * p* value	Cochran Q statistics (df)	Q *p* value
APOA1-DVT	8.11e−4 (7.07e−4)	0.284	22.23 (8)	0.004	25.89 (9)	0.002
APOB-DVT	0.001 (8.1e−4)	0.151	268.64 (17)	3.17e−47	304.36 (18)	6.07e−54
LDL-DVT	− 1.2e−4 (1.19e−4)	0.309	137.38 (67)	9.07e−07	139.53 (68)	7.42e−07
HDL-DVT	− 1.36e−5 (1.9e−4)	0.901	208.28 (84)	1.52e−12	208.32 (85)	2.39e−12
TGs-DVT	− 4.49e−5 (1.34e− 4)	0.740	125.44 (52)	5.22e−08	125.71 (53)	7.62e−08

*DVT* deep venous thrombosis, *APOA1* apolipoprotein A1, *APOB* apolipoprotein B, *LDL* low-density lipoprotein, *HDL* high-density lipoprotein, *TGs* triglycerides, *Se* standard error, *MR* Mendelian randomization, *IVW* inverse variance weighting.

The MR–Egger intercept in the analysis showed that there was no horizontal multiplicity (MR–Egger intercept *p* value = 0.284) (Table 2). The scatter plot shows the estimated impact of SNPs on exposure (APOA1) and outcome (DVT) (Fig. 1). The results of the “leave-one-out” test in the analysis showed that there was no abnormal IV in this analysis affecting the overall results (Supplementary File 3, Fig. S11). The forest plots and funnel plots in the analysis are shown in Supplementary File 3, Figs. S1 and S6. The funnel plot shows the location of directional horizontal pleiotropy in each result.

### The causal relationship between APOB and DVT

As shown in Table 1, based on the results of IVW (random effects), simple mode and MR–Egger methods, there was no evidence of a causal relationship between APOB and DVT (IVW (random effects): Beta = − 0.001, P_beta_ = 0.715; simple mode: Beta = 0.001, P_beta_ = 0.669; MR‒Egger: Beta = − 0.008, P_beta_ = 0.165). However, the results of the weighted median and weighted mode methods showed that APOB reduced the incidence of DVT (weighted median: Beta = − 0.004, P_beta_ = 0.005; weighted mode: Beta = − 0.006, P_beta_ = 0.003) (Table 1). The multivariable MR analysis also showed that there was no causal relationship between APOB and DVT (Beta = 0.008, P = 0.052) (Table 3).

**Table 3:** The results of multivariable MR.

Exposure-outcome	No. of SNPs	Beta	SE	*p* value
APOA1-DVT	7	− 0.002	0.004	0.701
APOB-DVT	9	0.008	0.004	0.052
LDL-DVT	60	− 0.008	0.003	0.012
HDL-DVT	73	7e−4	0.004	0.881
TGs-DVT	39	− 0.006	0.003	0.039

*DVT* deep venous thrombosis, *APOA1* apolipoprotein A1, *APOB* apolipoprotein B, *LDL* low-density lipoprotein, *HDL* high-density lipoprotein, *TGs* triglycerides, *Se* standard error, *MR* Mendelian randomization, *IVW* inverse variance weighting.

Because the heterogeneity test in the analysis indicated a certain level of heterogeneity (IVW: Q p values = 6.07e−54; MR–Egger: Q *p* values = 3.17e−47) (Table 2), we performed IVW (random effect) analysis, and the results showed that there was no causal relationship between APOB and DVT (Table 1). The MR–Egger intercept in the analysis showed that there was no horizontal multiplicity in the analysis (MR–Egger intercept *p* value = 0.151) (Table 2).

**Figure 2:**
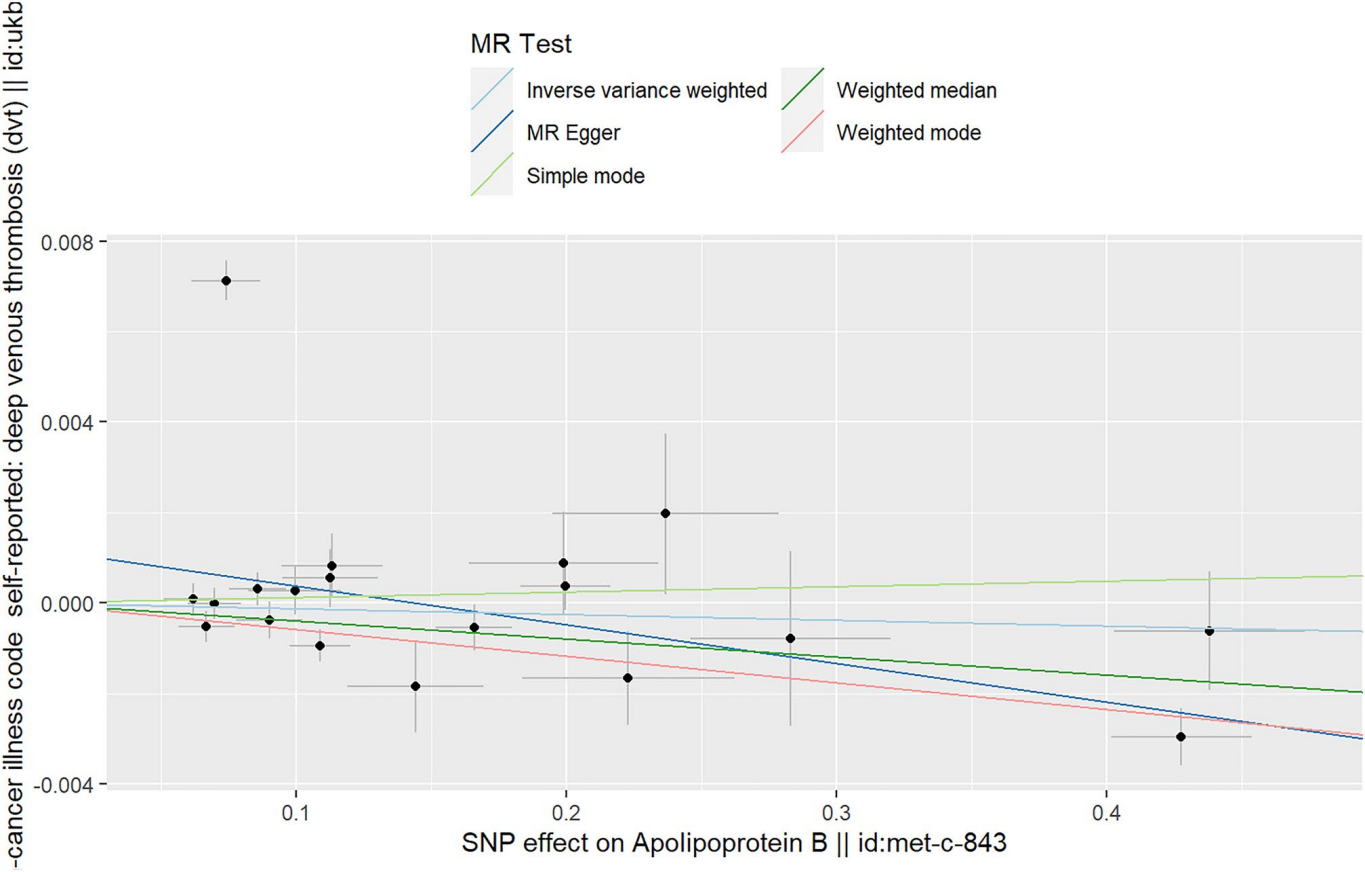
Scatter plot of the causal relationship between APOB and DVT. Analyses were conducted using the conventional IVW, weighted median, weighted mode, simple mode and MR‒Egger methods. The slope of each line corresponds to the estimated MR effect per method.

The scatter plot shows the estimated impact of SNPs on the exposure (APOB) and outcome (DVT) (Fig. 2). In addition, the results of the “leave-one-out” test in the analysis showed that there was no SNP affecting the overall results in this analysis (Supplementary File 3, Fig. S12). The forest plots and funnel plots in the analysis are shown in Supplementary File 3, Figs. S2 and S7.

### The causal relationship between LDL and DVT

As shown in Table 1, based on the results of IVW (random effects), weighted median, weighted mode, simple mode and MR‒Egger methods, there was no causal relationship between LDL and DVT (IVW (random effects): Beta = − 0.002, P_beta_ = 0.117; weighted median: Beta = − 0.001, P_beta_ = 0.366; weighted mode: Beta = − 0.001, P_beta_ = 0.637; simple mode: Beta = − 0.001, P_beta_ = 0.767; MR‒Egger: Beta = − 3e−4, P_beta_ = 0.881) (Table 1). The multivariable MR analysis also showed that there was no causal relationship between LDL and DVT (Beta = − 0.008, P = 0.012) (Table 3).

The heterogeneity analysis found some heterogeneity (IVW and MR–Egger Q *p* values < 0.05) (Table 2). The MR–Egger intercept in the analysis showed that there was no horizontal multiplicity (MR–Egger intercept *p* value = 0.309) (Table 2).

**Figure 3:**
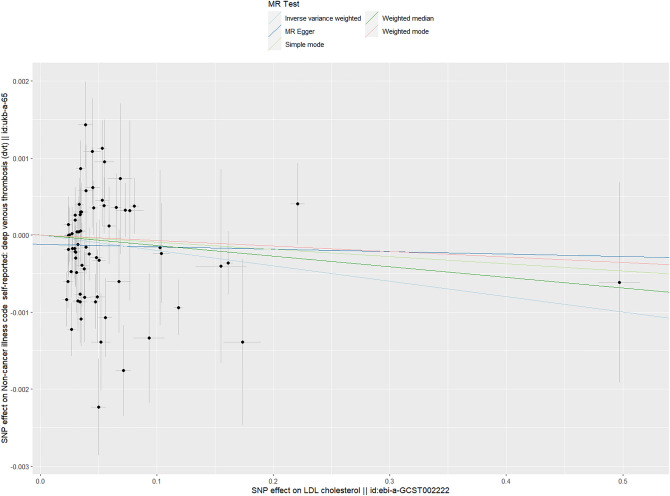
Scatter plot of the causal relationship between LDL and DVT. Analyses were conducted using the conventional IVW, weighted median, weighted mode, simple mode and MR‒Egger methods. The slope of each line corresponds to the estimated MR effect per method.

The scatter plot shows the estimated impact of SNPs on the exposure (LDL) and outcome (DVT) (Fig. 3). The results of the “leave-one-out” analysis showed that the results of the analyses were robust (Supplementary File 3, Fig. S13). The forest plots and funnel plots in the analysis are shown in Supplementary File 3, Figs. S3 and S8.

### The causal relationship between HDL and DVT

As shown in Table 1, in the analysis, we found that there was no causal relationship between HDL and DVT using various MR analysis methods (IVW (random effects): Beta = − 5e−4, P_beta_ = 0.670; weighted median: Beta = − 0.001, P_beta_ = 0.259; weighted mode: Beta = − 0.001, P_beta_ = 0.484; simple mode: Beta = 0.003, P_beta_ = 0.384; MR‒Egger: Beta = − 3.54–4, P_beta_ = 0.870). The multivariable MR analysis also showed that there was no causal relationship between HDL and DVT (Beta = 7e−4, P = 0.881) (Table 3).

The heterogeneity test in the analysis found that there was some heterogeneity (the Q *p* values of IVW and MR–Egger methods were both less than 0.05). The MR–Egger intercept in the analysis showed that there was no horizontal multiplicity (MR–Egger intercept *p* value = 0.901) (Table 2).

**Figure 4:**
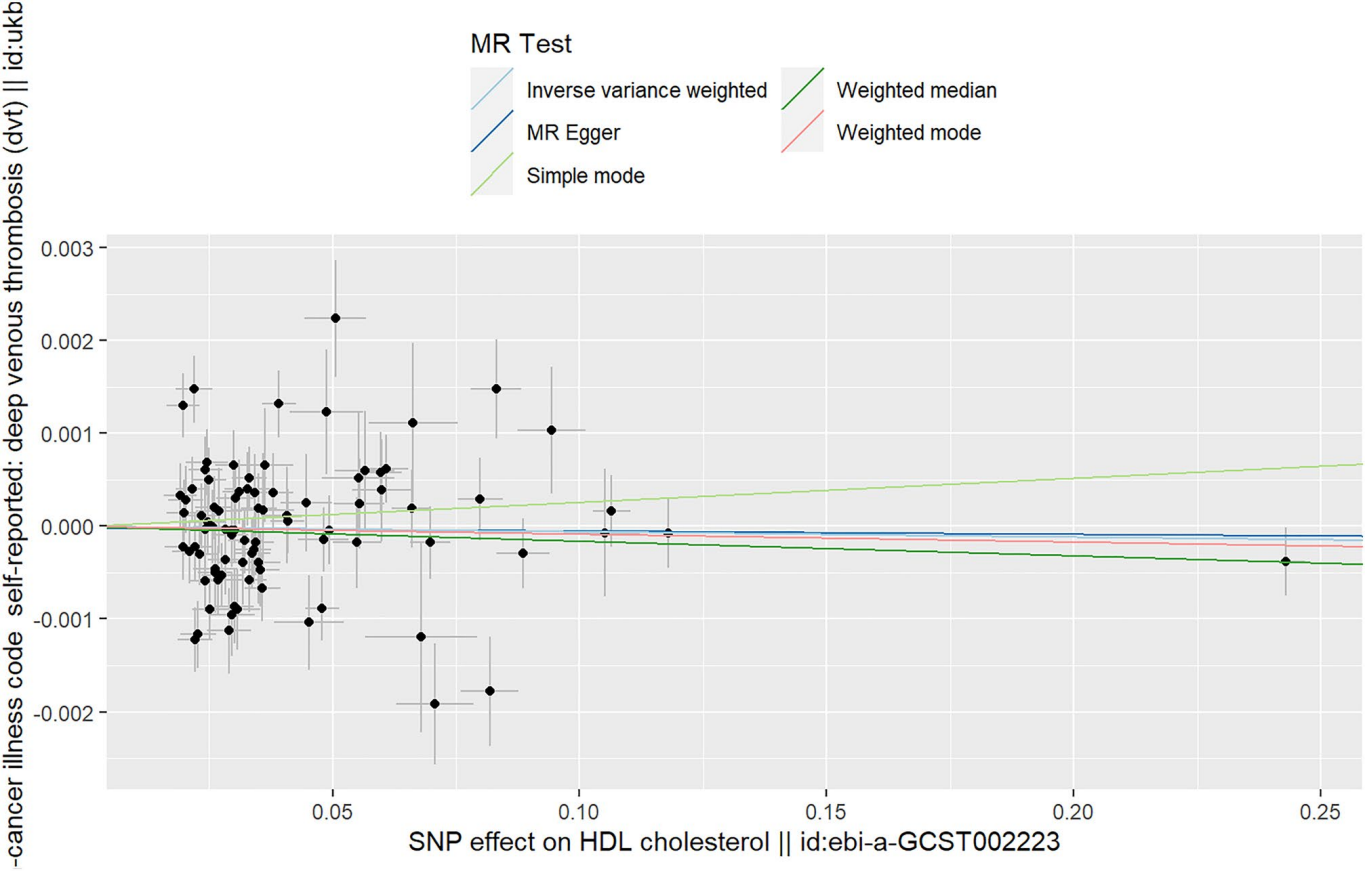
Scatter plot of the causal relationship between HDL and DVT. Analyses were conducted using the conventional IVW, weighted median, weighted mode, simple mode and MR‒Egger methods. The slope of each line corresponds to the estimated MR effect per method.

The scatter plot shows the estimated impact of SNPs on the exposure (HDL) and outcome (DVT) (Fig. 4). The results of the “leave-one-out” analysis showed that there was no abnormal IV in this analysis affecting the overall results (Supplementary File 3, Fig. S14). The forest plots and funnel plots in the analysis are shown in Supplementary File 3, Figs. S4 and S9.

### The causal relationship between TGs and DVT

Based on the results of IVW (random effects), weighted median and weighted mode, methods we found a suggested negative causal relationship between TGs and DVT (IVW (random effects): Beta = − 0.004, P_beta_ = 0.038; weighted median: Beta = − 0.004, P_beta_ = 0.017; weighted mode: Beta = − 0.003, P_beta_ = 0.038) (Table 1). However, based on the results of simple mode and MR‒Egger methods, there was no causal relationship between TGs and DVT (simple mode: Beta = − 0.003, P_beta_ = 0.270; MR‒Egger: Beta = − 0.003, P_beta_ = 0.335). The multivariable MR analysis also showed that there was no causal relationship between TGs and DVT (Beta = − 0.006, P = 0.039) (Table 3).

**Figure 5:**
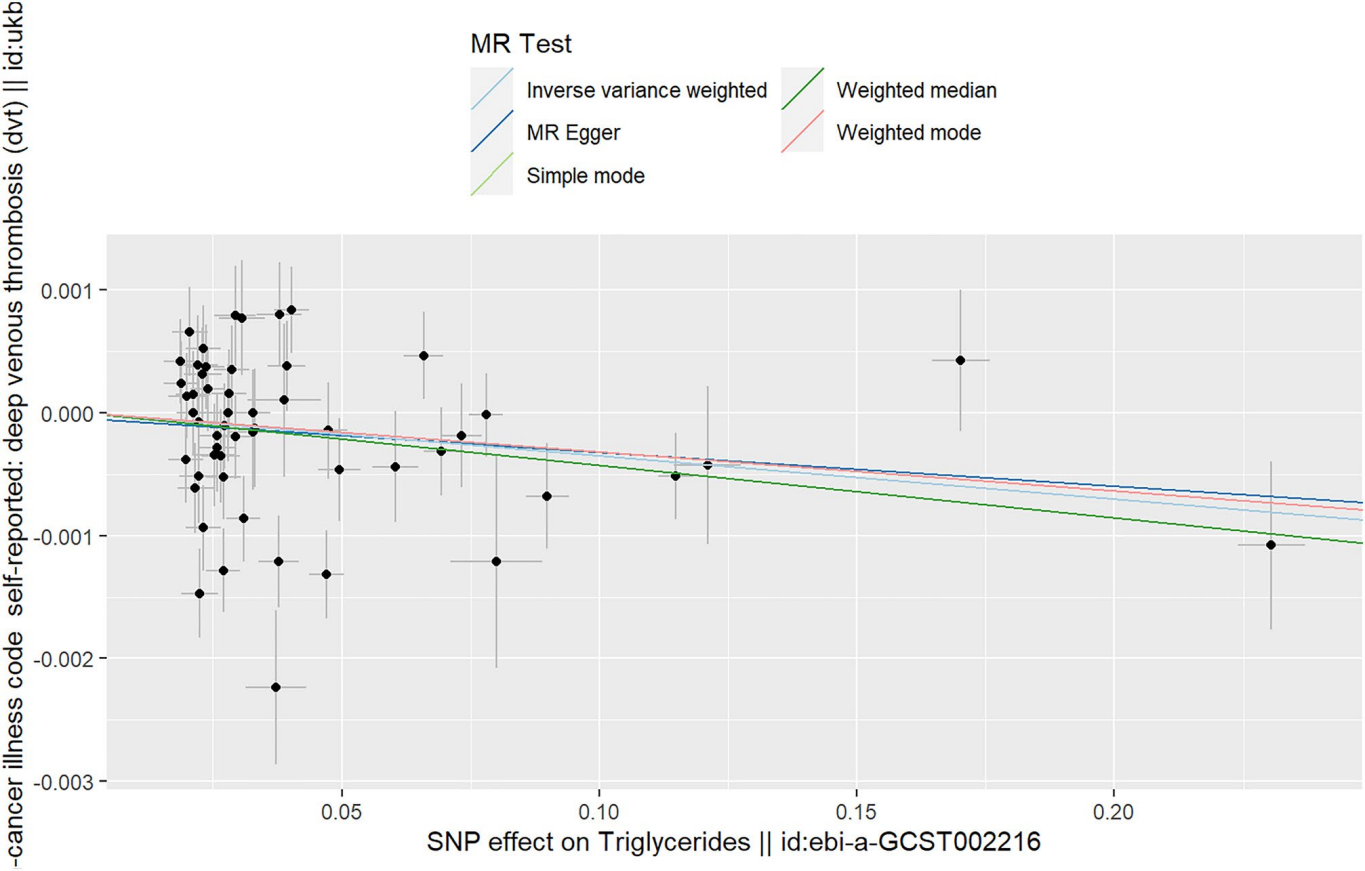
Scatter plot of the causal relationship between TGs and DVT. Analyses were conducted using the conventional IVW, weighted median, weighted mode, simple mode and MR‒Egger methods. The slope of each line corresponds to the estimated MR effect per method.

The scatter plot shows the estimated impact of SNPs on the exposure (TG) and outcome (DVT) (Fig. 5). The heterogeneity test showed some heterogeneity (IVW and MR–Egger Q value < 0.05). The MR–Egger intercept in the analysis showed that there was no horizontal multiplicity (MR–Egger intercept *p* value > 0.05) (Table 2).

The results of the “leave-one-out” method test in the analysis showed that no abnormal IV in the analyses affected the overall results (Supplementary File 3, Fig. S15). The forest plots and funnel plots in the analysis are shown in Supplementary File 3, Figs. S5 and S10.

## Discussion

In this study, we used two-sample Mendelian randomization to analyse summary GWAS data and found that our two-sample MR analysis failed to detect a statistically significant causal relationship between five common circulating lipids and DVT. This result differs from those of previous clinical studies. For example, Morelli et al. found that lower levels of APOA1 and APOB were associated with an increased risk of DVT^4^. In addition, Sabine et al. found that patients with high levels of APOA1 and HDL had a lower risk of recurrent DVT^24^. Petter et al. found no association between the risk of DVT and total cholesterol, low-density lipoprotein cholesterol, high-density lipoprotein cholesterol, triglycerides, glucose or smoking^25^. In our previous study^26^, we found that there was no significant causal relationship between three traditional lipids (LDL, HDL and TGs) and VTE (DVT and PE) from a genetic point of view. The GWAS data used in our current Mendelian randomization study are very different from those used in previous studies, and we used new GWAS data to further prove that there is no causal relationship between LDL, HDL, TGs and DVT. In addition, this study found that there was no causal relationship between APOA1 and APOB and DVT, which adds new evidence to the study of the causal relationship between blood lipids and DVT.

Compared with major lipids, there are few reports on the relationship between DVT and APOA1 and APOB levels. Apolipoprotein was not associated with the risk of DVT in cohort studies^7,27^. In other clinical studies, these apolipoprotein-DVT associations have been limited to certain subgroups; for example, in a case–control study that included only men, low levels of APOA1 were associated with an increased risk of DVT^28^. High levels of APOA1 in the Women's Health Study were associated with an increased risk of blood clots in hormone users^29^. In another small case–control study, high APOB levels appeared to increase the risk of DVT in men^30^. These contradictory results may be related to the adjustment for potential confounding factors, as the MR method can reduce confounding factors in observational studies. Our study showed that APOA1 and APOB had no significant causal relationship with DVT from a genetic perspective.

Statins and fibrates are commonly used to reduce blood lipids. A meta-analysis also found that statins may reduce the risk of DVT, whereas fibrates may increase this risk^31^. The authors did not provide an effective explanation for this result, but our results showed that TGs had no protective effect on DVT. Therefore, well-designed in vitro and in vivo studies are strongly encouraged, and DVT events should be included as a major end point in new clinical studies of fibrates. In recent years, an increasing number of studies have found that statins can effectively reduce the incidence of DVT^32,33^; for example, rosuvastatin significantly reduces the incidence of symptomatic venous thromboembolism^34^, and in vitro and observational studies have found that statins may have beneficial effects on blood vessel walls and antithrombotic formation^35,36^. A meta-analysis found that the use of statins and antiplatelet therapy was associated with a significant reduction in the incidence of venous thromboembolism^37^. It is well known that statins not only have lipid-lowering effects but also have a variety of vascular protective effects that are independent of changes in cholesterol levels. These effects are attributed to the anti-inflammatory and antithrombotic properties of statins, which can alter endothelial dysfunction and regulate angiogenesis^38^. Therefore, the documentation that statins can effectively reduce the incidence of DVT does not conflict with our results. However, there are also contrary findings about the efficacy of statins in preventing DVT^39^. Therefore, more clinical and basic studies are needed to explore whether statins can prevent DVT at this stage.

In this study, we selected SNPs with a genome-wide association and independent inheritance without any LD as IVs to detect a causal relationship between circulating lipids (APOA1, APOB, LDL, HDL and TGs) and DVT. To make our conclusions more robust and reliable, we used several analysis methods for comparison and verification. The F value in each analysis was greater than 10, which effectively avoided the weak tool variable bias. Of course, our analysis was somewhat restrictive. First, because our analysis was based on publicly available aggregate data, the data provided did not allow us to conduct other subgroup analyses to address associations with specific factors such as age, sex and other DVT risk factors. Second, this study was limited to participants of European origin. Therefore, our results may not apply to people of other races. Finally, regarding the results that were not statistically significant in this study, we could not completely rule out an association between these circulating lipids and DVT because this may reflect the small sample size and the lack of statistical power of the MR analysis.

## Conclusions

Based on our results, our two-sample MR analysis failed to detect a statistically significant causal relationship between five common circulating lipids and DVT. Subsequent studies or MR analysis based on more genetic instruments are needed to validate these findings and clarify the potential mechanism of the effect of circulating lipids on the development of DVT.

## Supplementary Information

Supplementary Information 1.

Supplementary Information 2.

Supplementary Information 3.

## Data Availability

The data used in the present study are all publicly available at https://gwas.mrcieu.ac.uk/.

## Abbreviations

DVT: Deep venous thrombosis
VT: Venous thrombosis
SNPs: Single-nucleotide polymorphisms
GWAS: Genome-wide association study
IVW: Inverse variance weighting
LDL: Low-density lipoprotein
HDL: High-density lipoprotein
TGs: Triglycerides
APOB: Apolipoprotein B
APOA1: Apolipoprotein A1
IV: Instrumental variable
ORs: Odds ratios
LD: Linkage disequilibrium
CIs: Confidence intervals
