# Supplementary material for: A two-sample Mendelian randomization study of circulating lipids and deep venous thrombosis
Source: Sci Rep. 2023 May 8;13:7432. doi: 10.1038/s41598-023-34726-3 (PMC10167313; doi:10.1038/s41598-023-34726-3)
Supplement: Supplementary file 1 — Supplementary Information 1. [file 41598_2023_34726_MOESM1_ESM.docx]

Table S1. Characteristics of datasets.

| Trait | GWAS ID | Sample Size | Number of SNPs | Population |
| --- | --- | --- | --- | --- |
| APOA1 | met-c-842 | 20,687 | 11,760,646 | European |
| APOB | met-c-843 | 20,690 | 11,813,266 | European |
| LDL | ebi-a-GCST002222 | 94,595 | 2,409,690 | European |
| HDL | ebi-a-GCST002223 | 94,595 | 2,418,527 | European |
| TG | ebi-a-GCST002216 | 94,595 | 2,410,057 | European |
| DVT | ukb-a-65 | 337,159 | 10,894,596 | European |

Note: APOA1, Apolipoprotein A1; APOB, Apolipoprotein B; HDL, high-density lipoprotein cholesterol; LDL, low-density lipoprotein cholesterol; TG, triglycerides; DVT, Deep venous thrombosis
