# Supplementary material for: A two-sample Mendelian randomization study of circulating lipids and deep venous thrombosis
Source: Sci Rep. 2023 May 8;13:7432. doi: 10.1038/s41598-023-34726-3 (PMC10167313; doi:10.1038/s41598-023-34726-3)
Supplement: Supplementary file 3 — Supplementary Information 3. [file 41598_2023_34726_MOESM3_ESM.docx]

**Supplemental Figure S1: Forest plot of variant specific inverse variance estimates for the causal association between APOA1 and DVT**

**
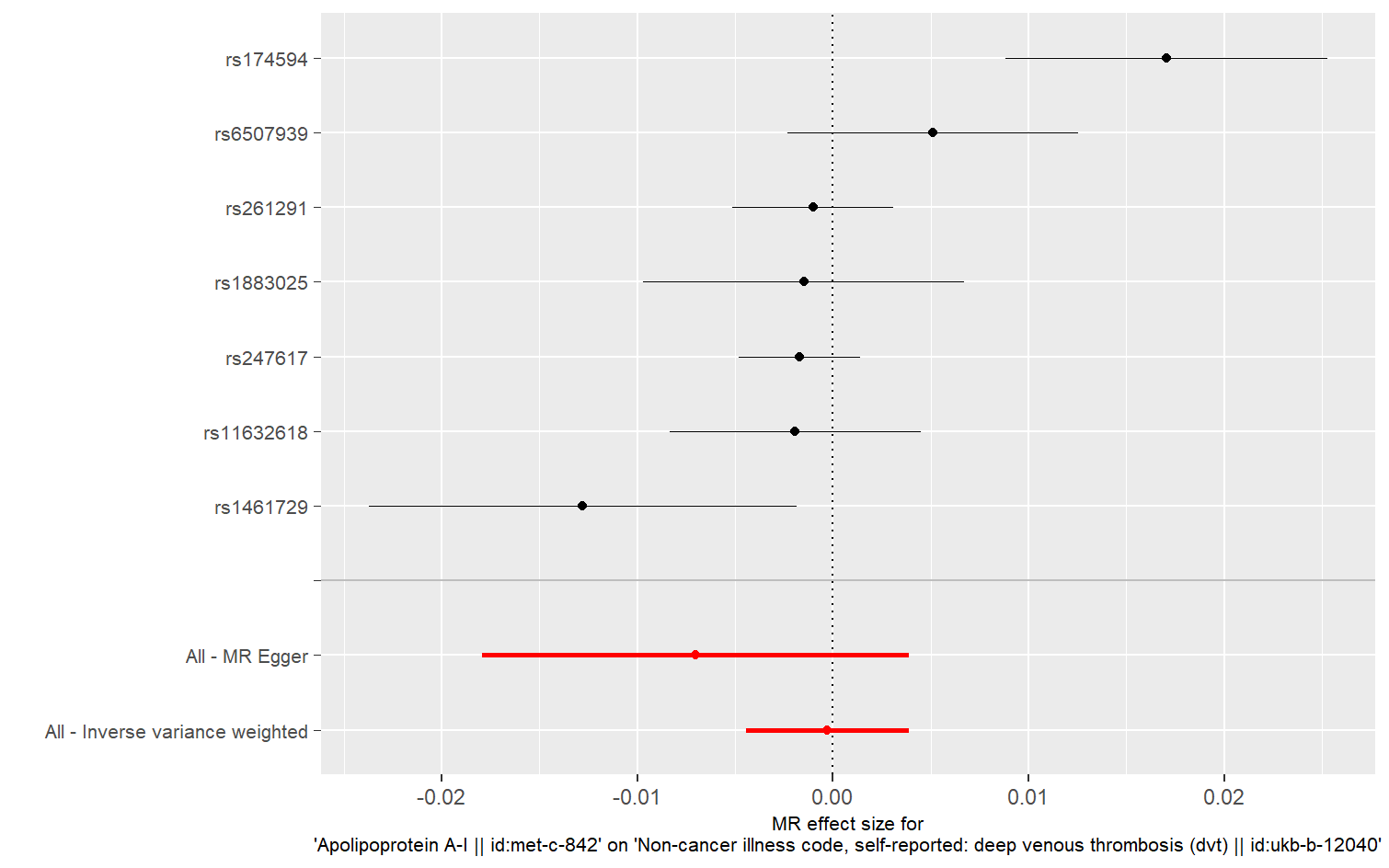
**

**Supplemental Figure S2: Forest plot of variant specific inverse variance estimates for the causal association between APOB and DVT**

**
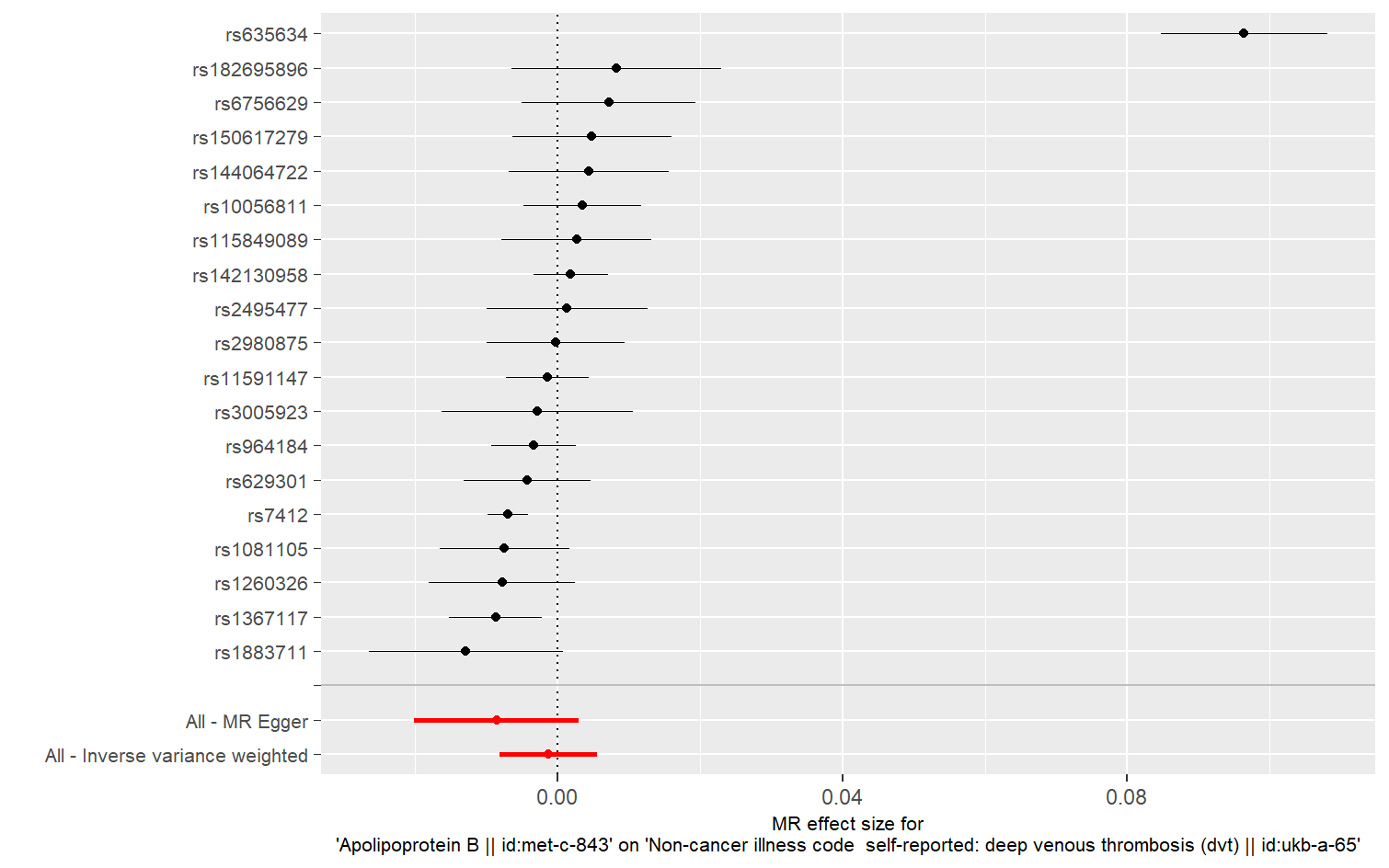
**

**Supplemental Figure S3: Forest plot of variant specific inverse variance estimates for the causal association between LDL and DVT**

**
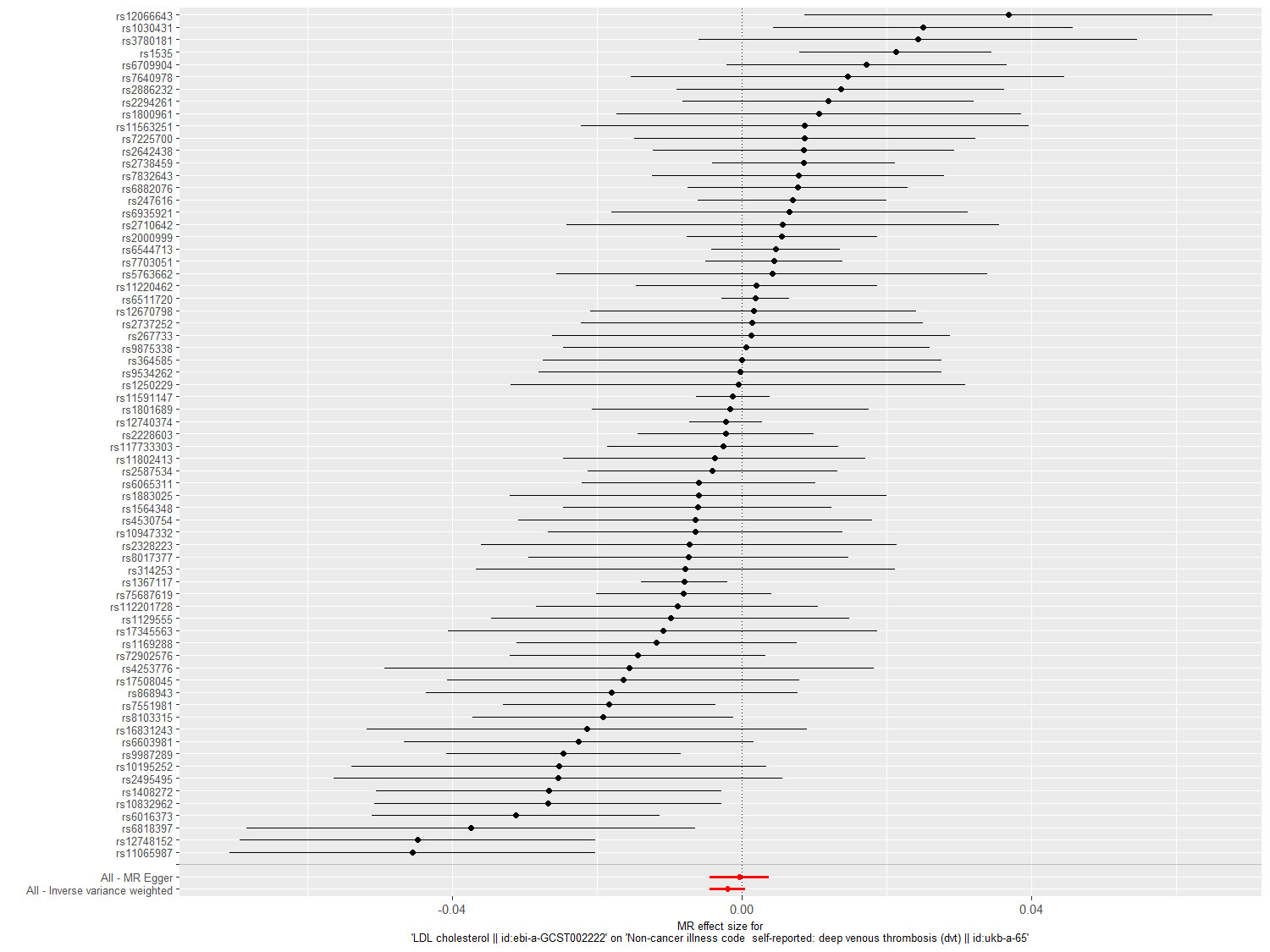
**

**Supplemental Figure S4: Forest plot of variant specific inverse variance estimates for the causal association between HDL and DVT**

**
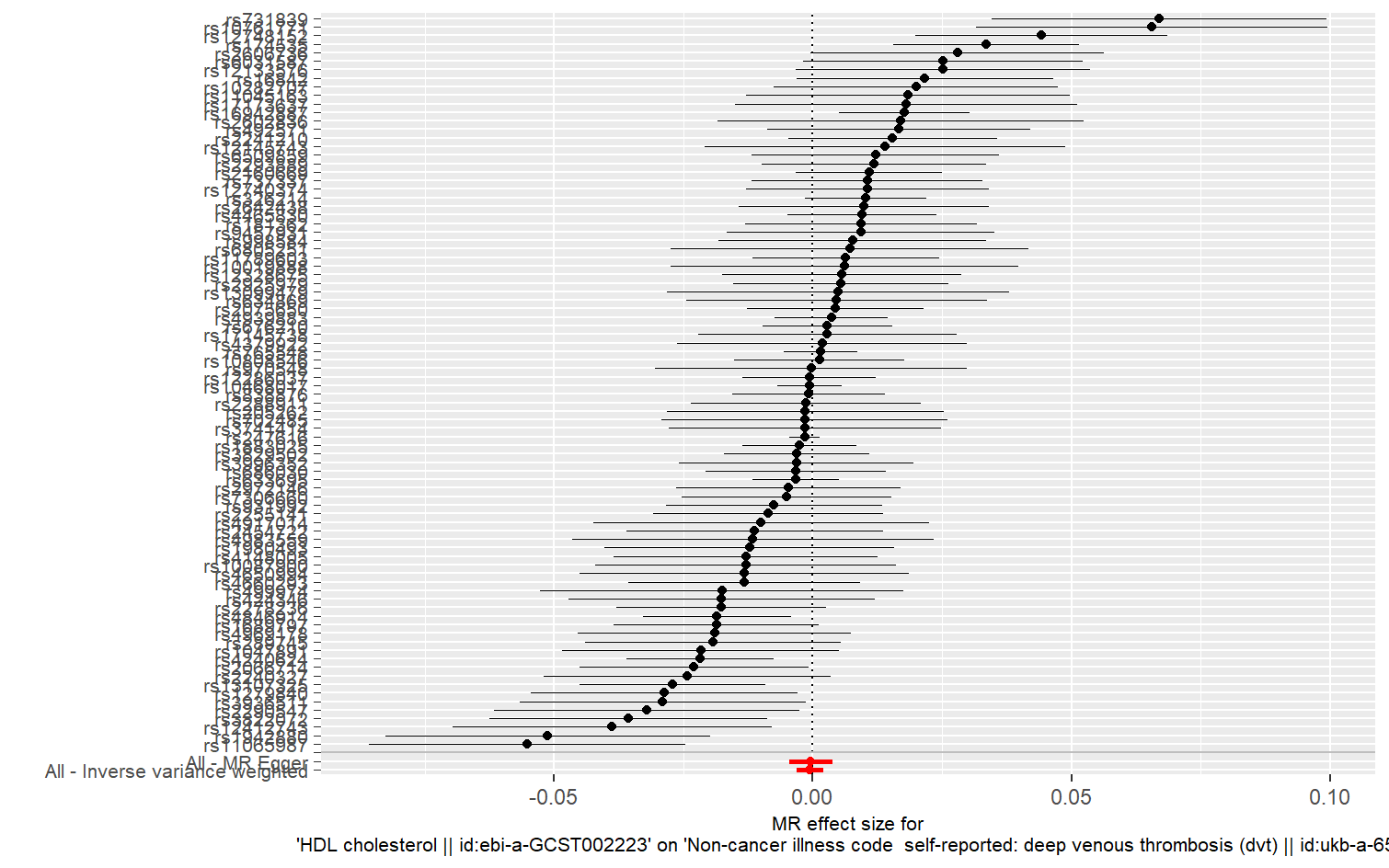
**

**Supplemental Figure S5: Forest plot of variant specific inverse variance estimates for the causal association between TG and DVT**

**
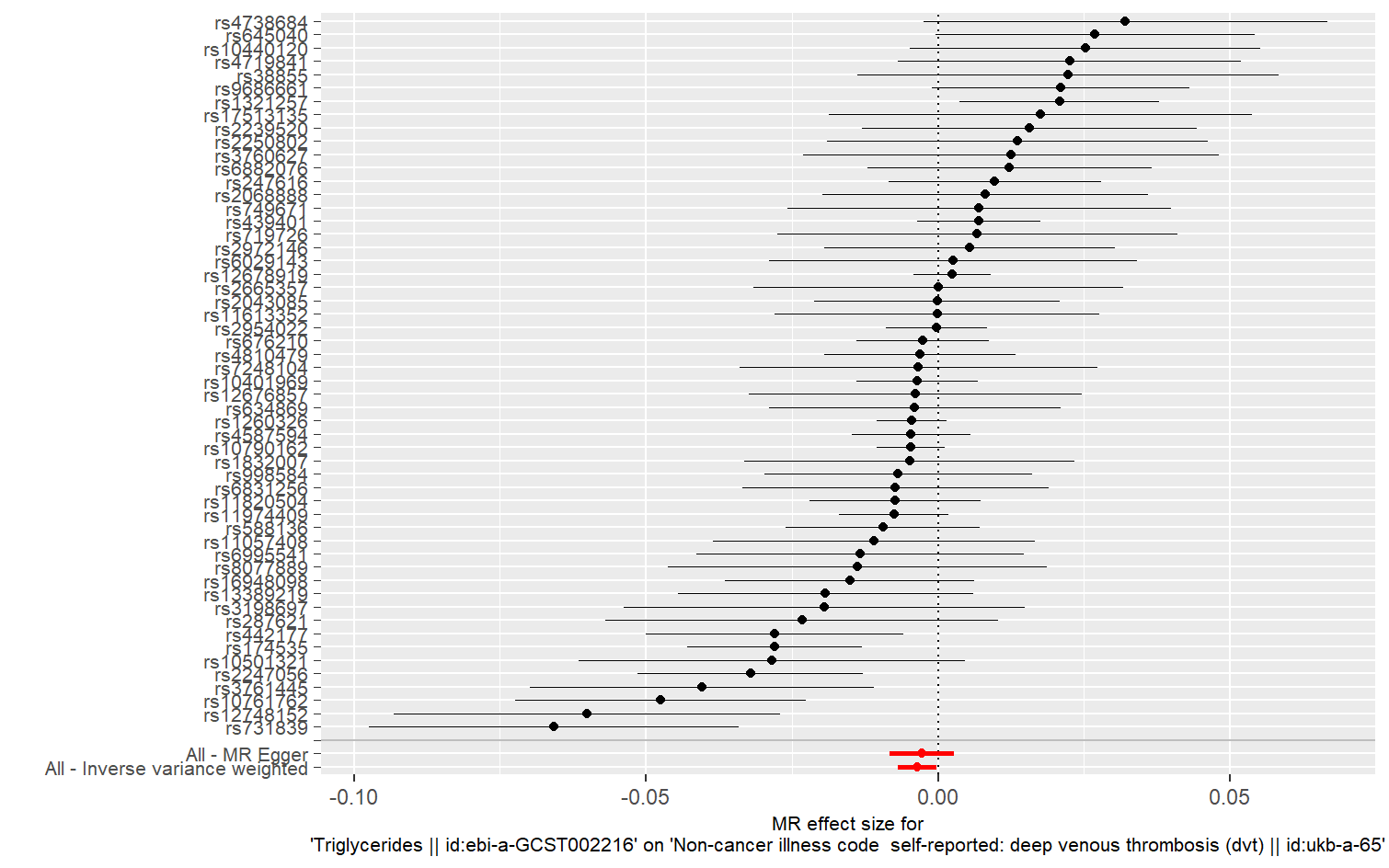
**

**Supplemental Figure S6: Funnel plot of the causal association between APOA1 and DVT**

**
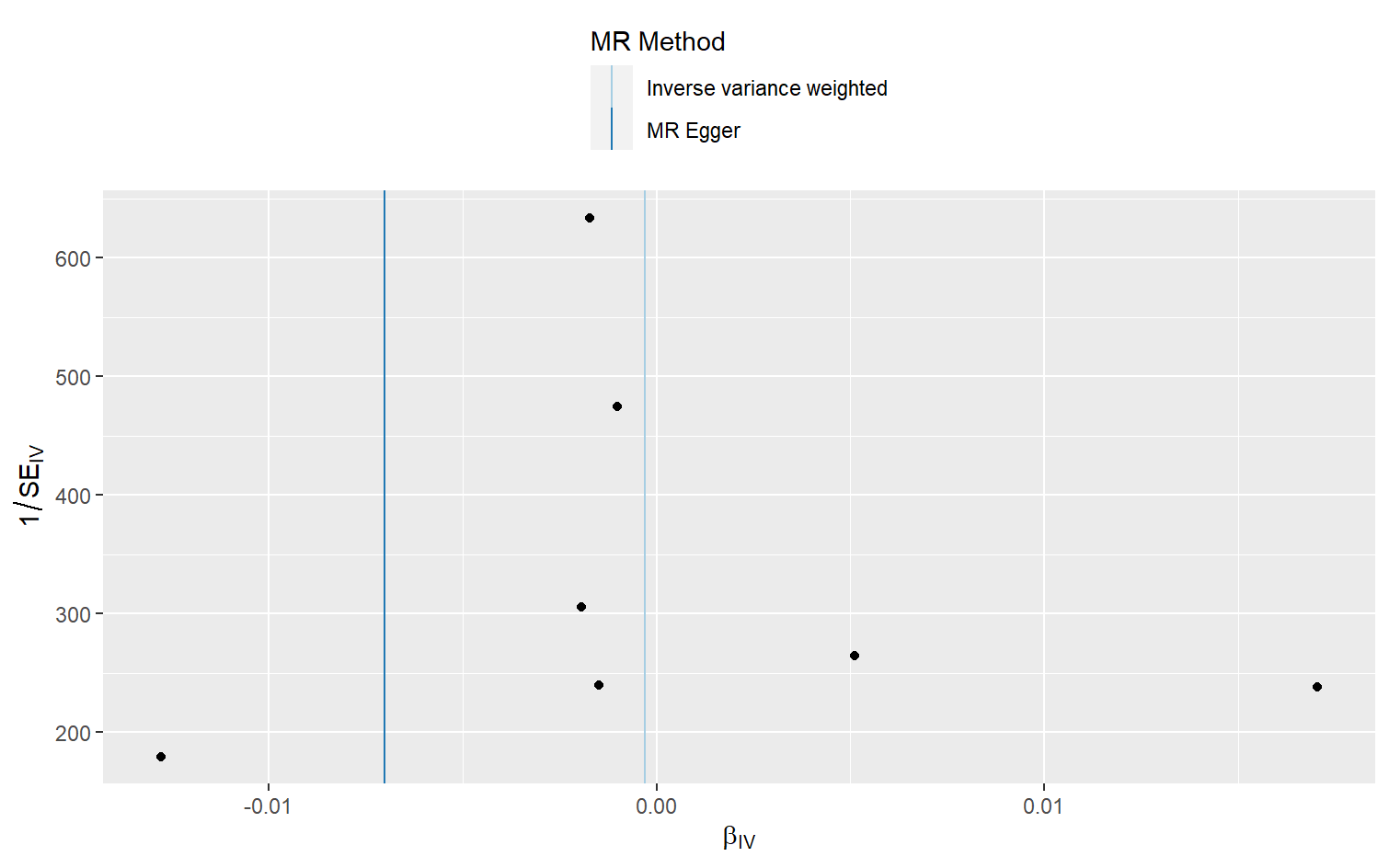
**

**Supplemental Figure S7: Funnel plot of the causal association between APOB and DVT**

**
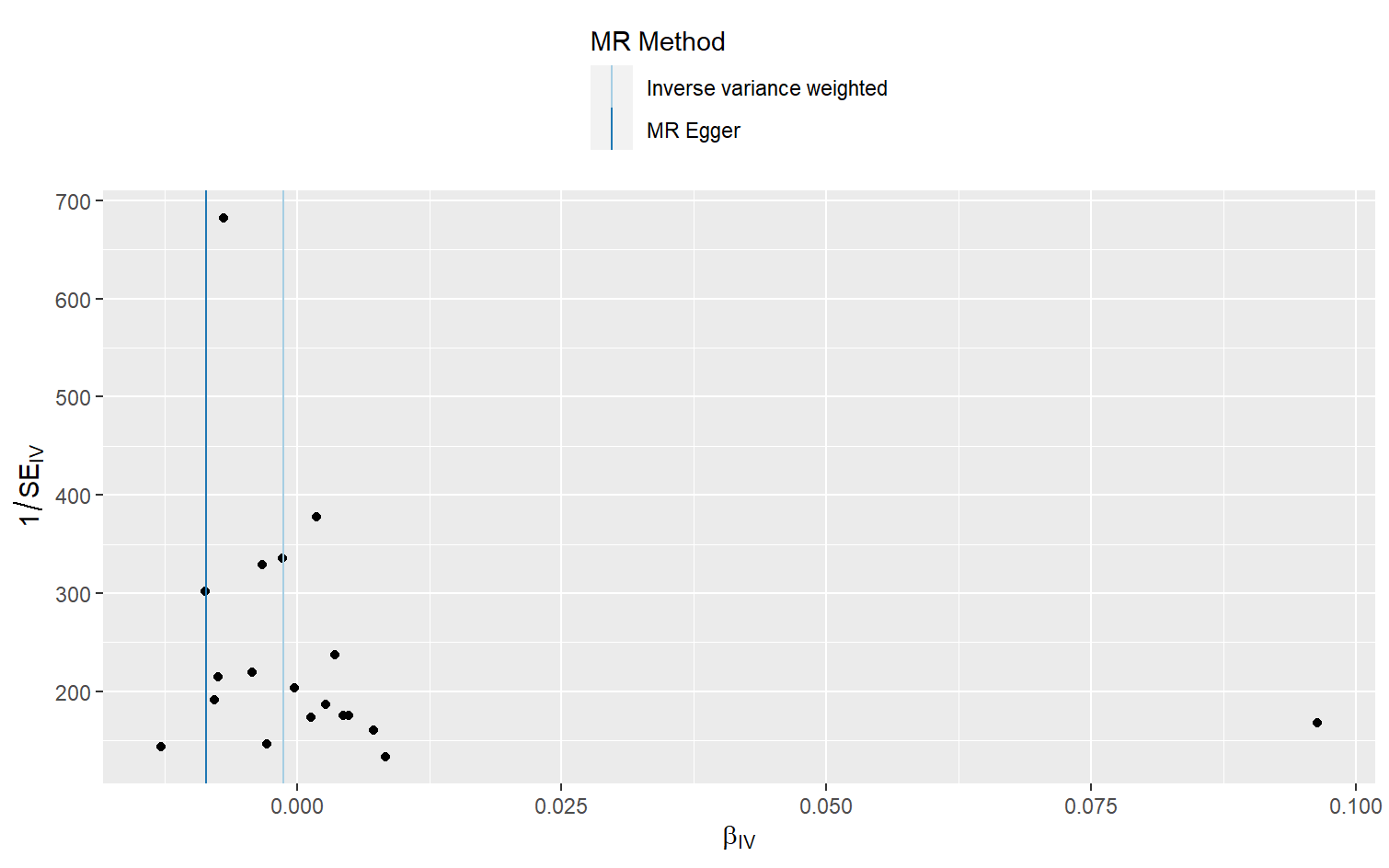
**

**Supplemental Figure S8: Funnel plot of the causal association between LDL and DVT**

**
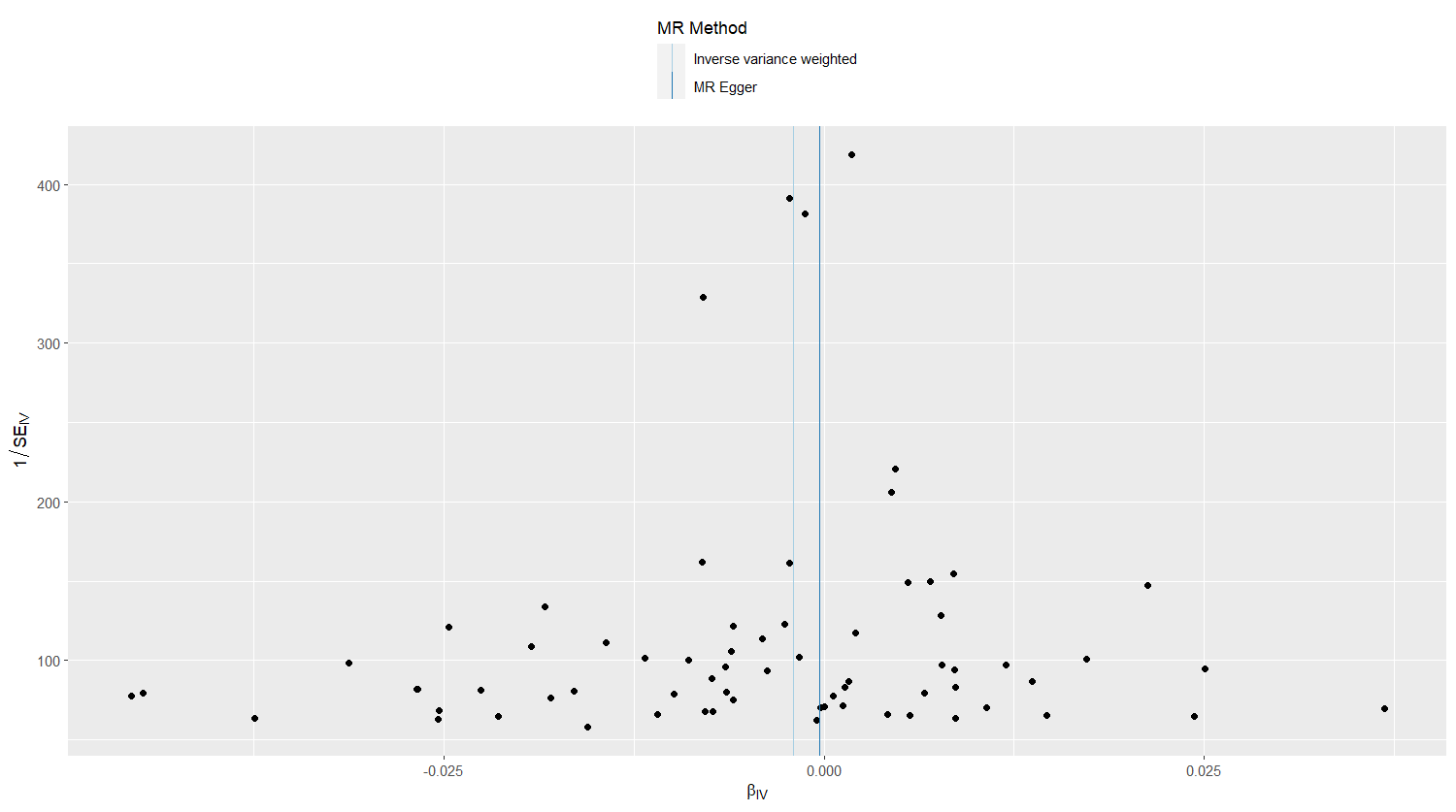
**

**Supplemental Figure S9: Funnel plot of the causal association between HDL and DVT**

**
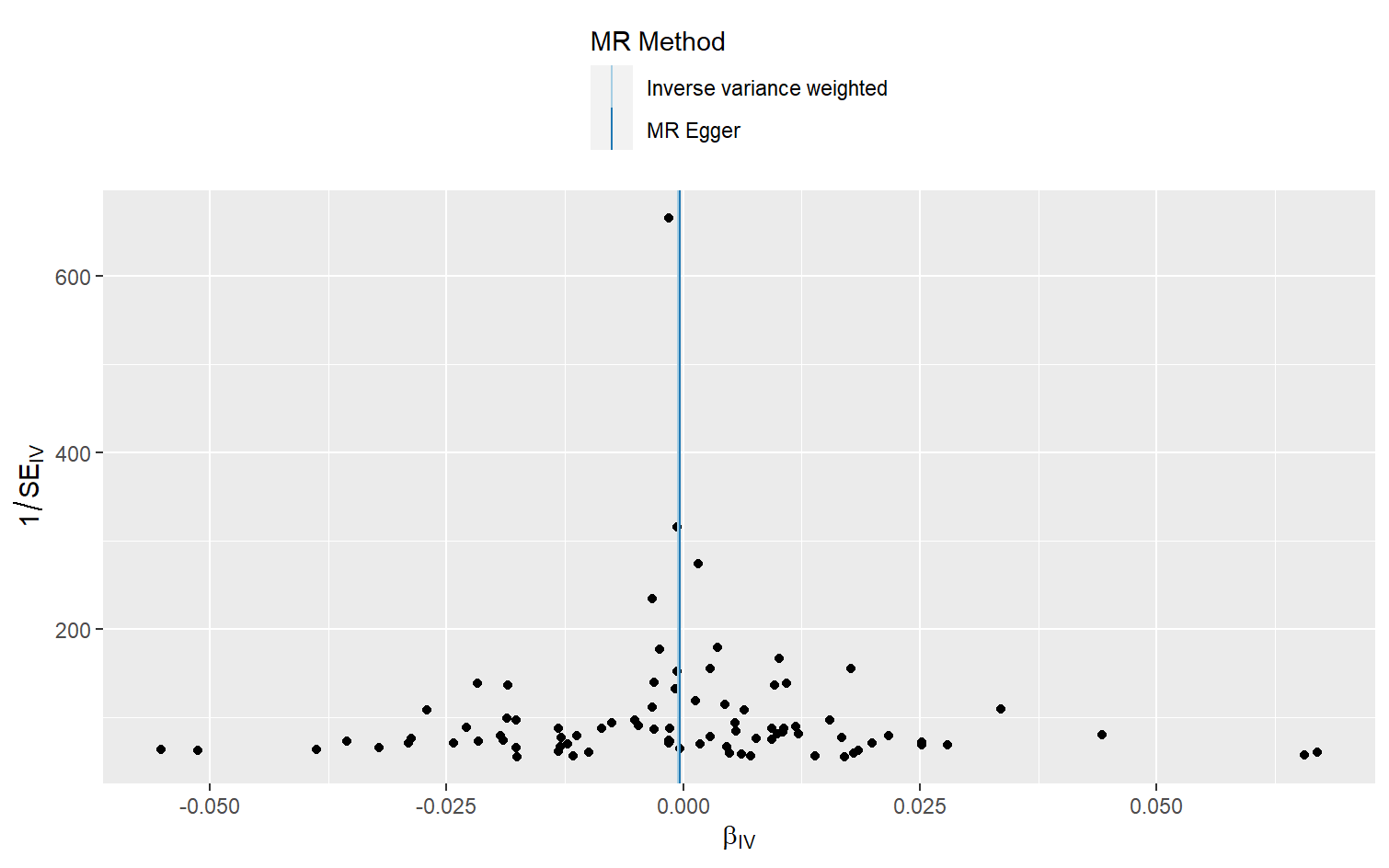
**

**Supplemental Figure S10: Funnel plot of the causal association between TG and DVT**

**
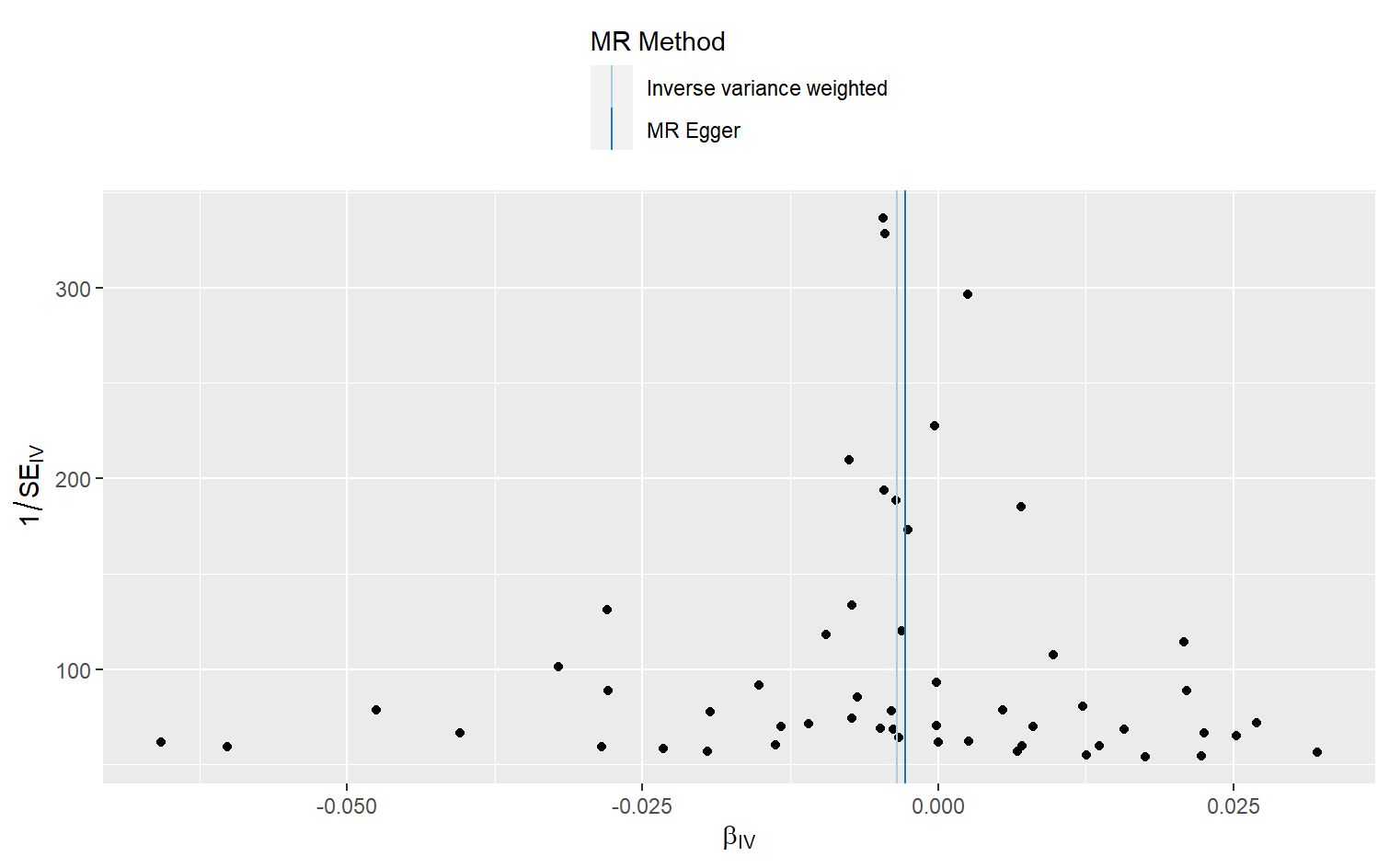
**

**Supplemental Figure S11: Leave-one-out test plot of the causal association between APOA1 and DVT**

**
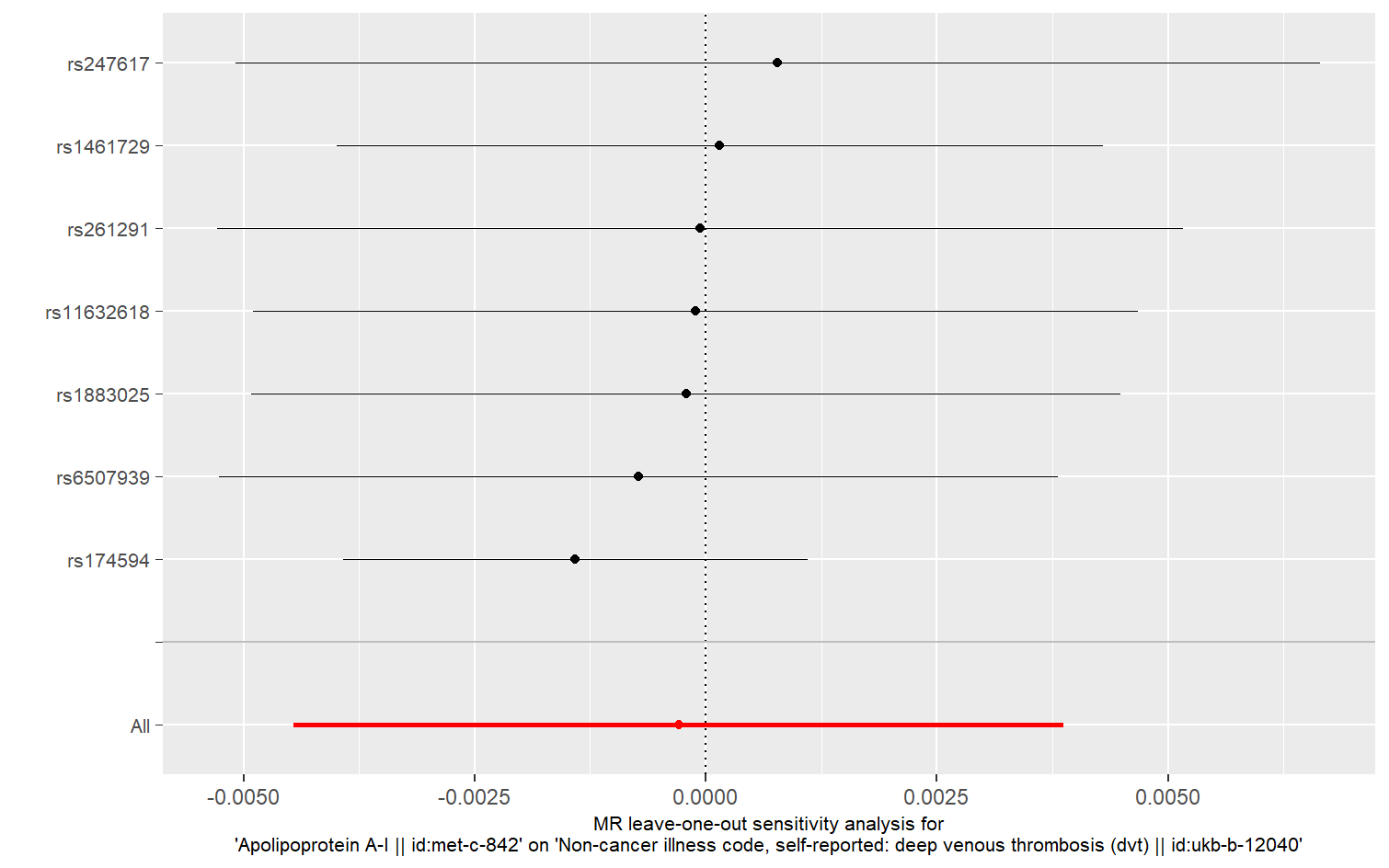
**

**Supplemental Figure S12: Leave-one-out test plot of the causal association between APOB and DVT**

**
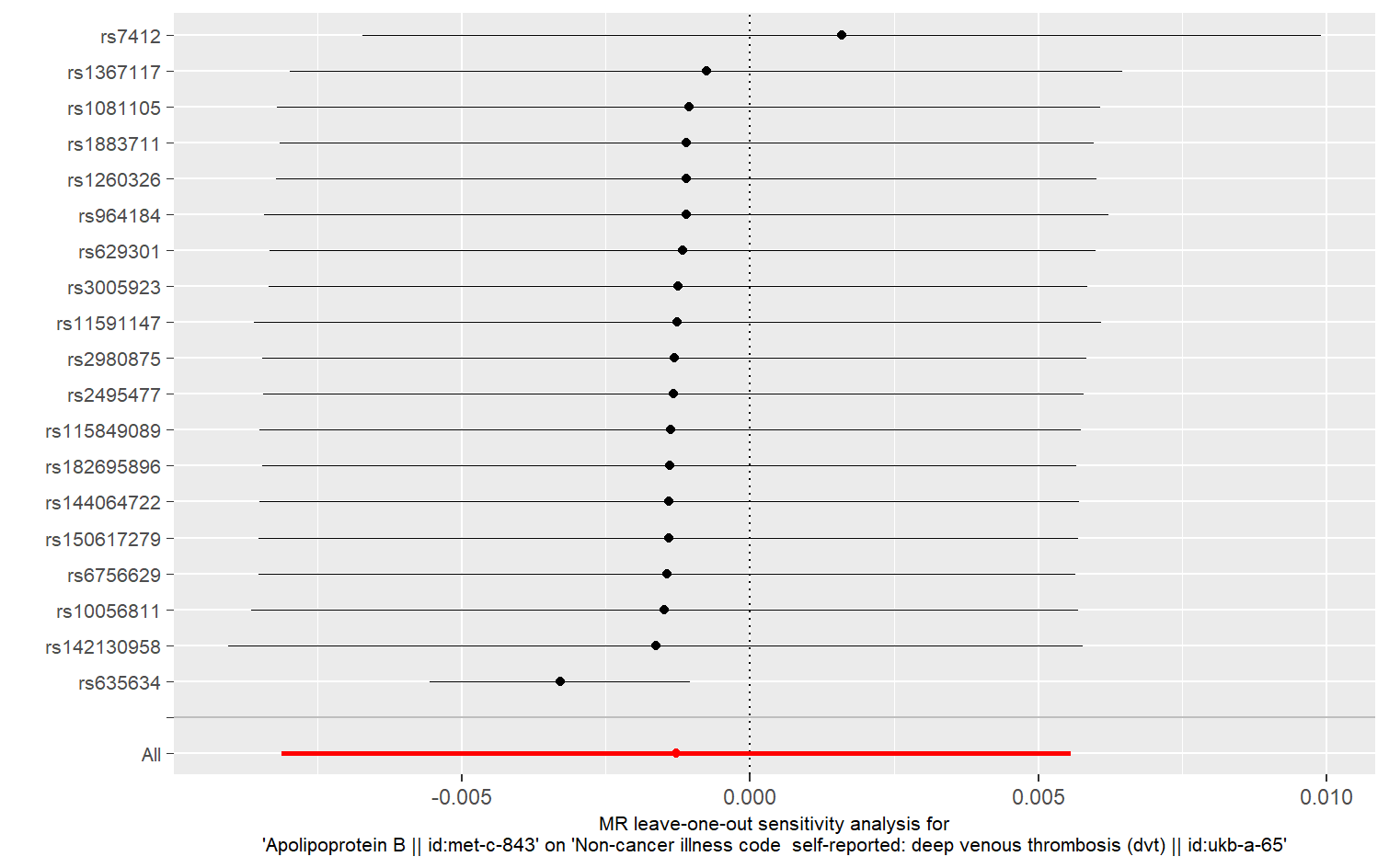
**

**Supplemental Figure S13: Leave-one-out test plot of the causal association between LDL and DVT**

**
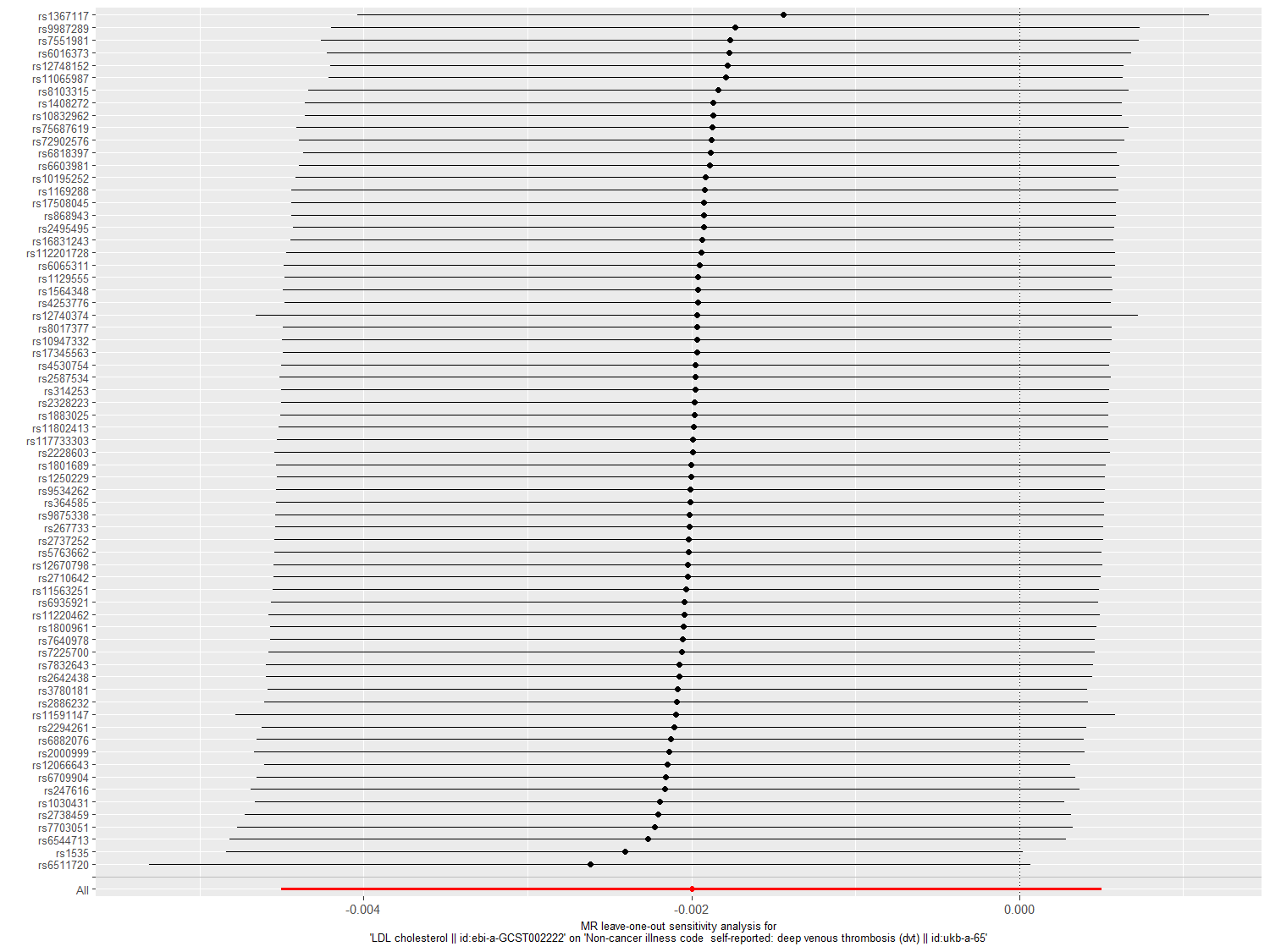
**

**Supplemental Figure S14: Leave-one-out test plot of the causal association between HDL and DVT**

**
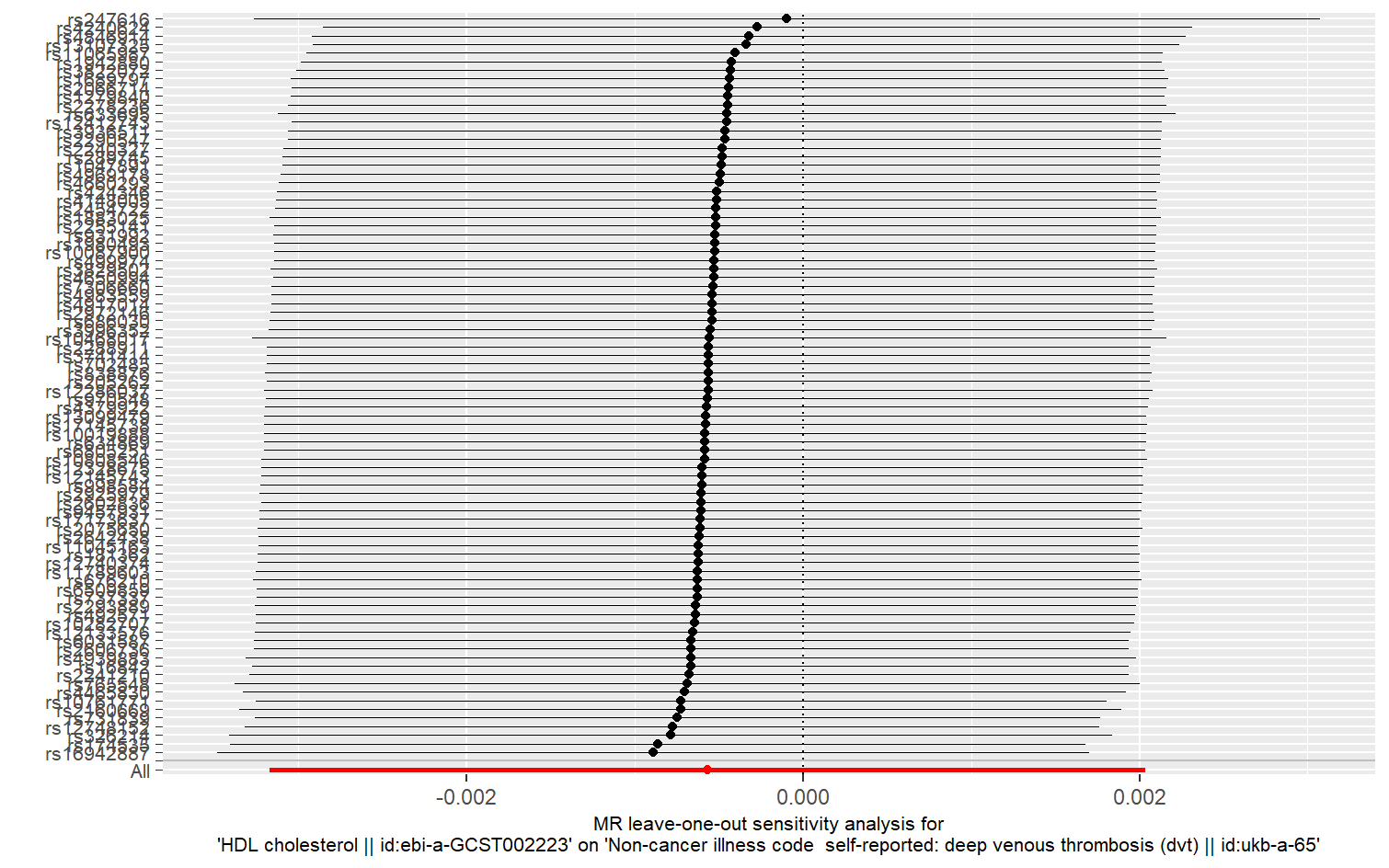
**

**Supplemental Figure S15: Leave-one-out test plot of the causal association between TG and DVT**

**
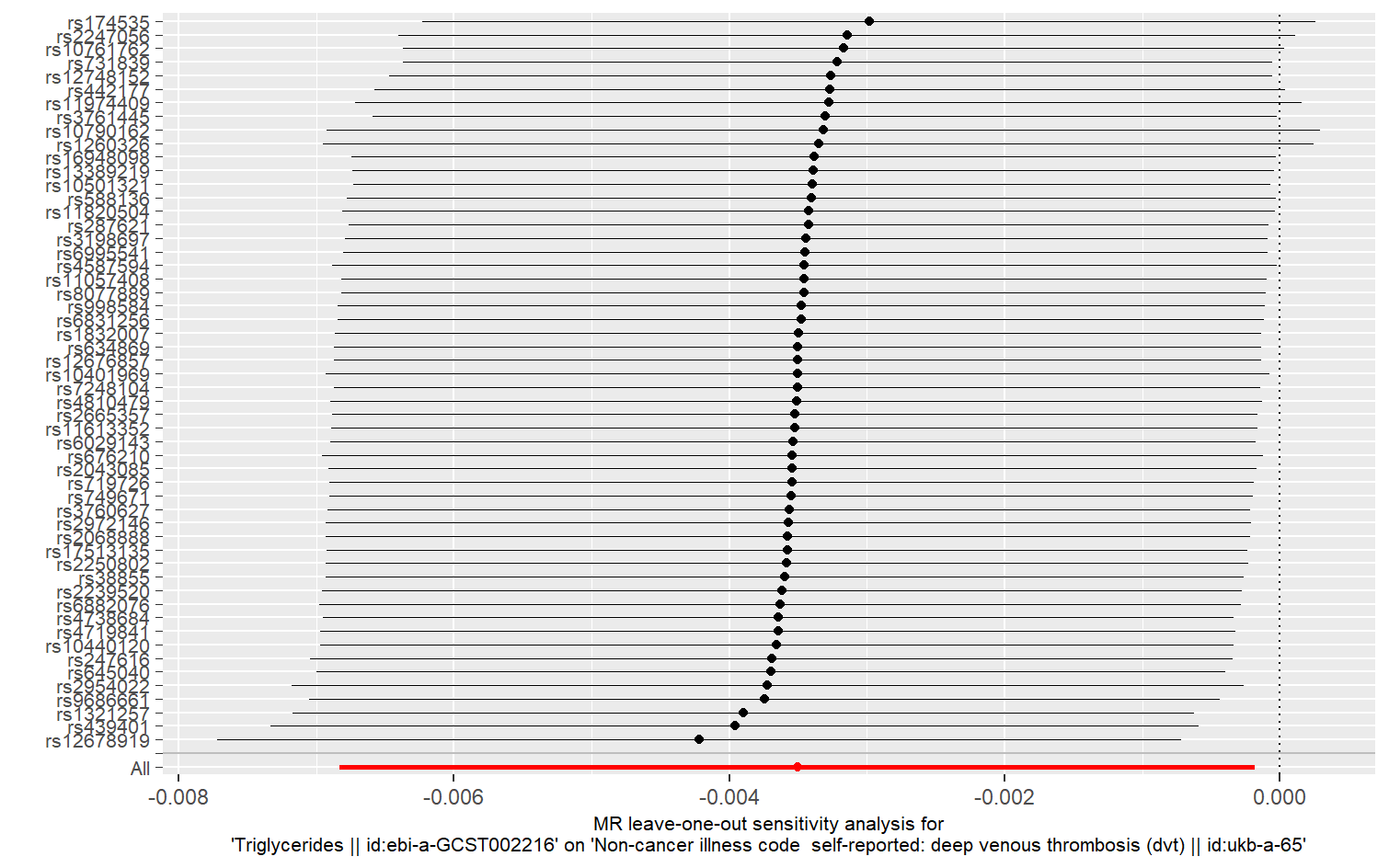
**
